# Radiation Recall Dermatitis following Radioactive Iodine Therapy: A New Observation

**DOI:** 10.1155/2021/8422748

**Published:** 2021-10-22

**Authors:** Ikechukwu Chidobem, Tahereh Orouji Jokar, Chisom Mgbodile, Francis Mgbodile, Ghassan Bassil, Nazia Khan

**Affiliations:** St. Mary's General Hospital, Passaic, NJ, USA

## Abstract

A 47-year-old female, who had previously received adjuvant right breast radiation for ductal carcinoma in situ, presented with right breast edema, erythema, and pain. This developed about two and a half weeks following radioactive iodine therapy for thyroid carcinoma. A biopsy was performed to rule out malignancy, since inflammatory breast cancer can present with similar symptoms. This confirmed radiation recall dermatitis (RRD) as the most likely diagnosis. RRD is an inflammatory reaction occurring in a previously irradiated field and was first described in 1959. Subsequent reports in the literature have associated it with the administration of other drugs, mostly chemotherapy. To our knowledge, this is the first reported case of RRD following radioactive iodine therapy.

## 1. Introduction

Radiation recall dermatitis (RRD) is a poorly understood phenomenon characterized by the development of an inflammatory skin reaction in a previously quiescent radiation field, representing the “recalling” of an effect similar in appearance to that of an acute radiation reaction, following the administration of a triggering agent [1, 2]. RRD typically occurs within days to weeks of exposure to the precipitating drug, and the interval from irradiation can range from days to several years [3–5]. To our knowledge, radiation recall dermatitis following radioactive iodine therapy has not been previously reported in the literature.

## 2. Case Presentation

A 47-year-old female was found to have two masses in the right breast following a screening mammogram, as well as subsequent breast ultrasound and MRI. Ultrasound-guided core biopsies showed a benign fibroadenoma, as well as a fibroadenoma with associated atypical lobular hyperplasia, apocrine metaplasia, and microcalcifications. Excisional breast biopsy showed an ER-positive fibroadenoma with involvement by foci of ductal carcinoma in situ within less than one millimeter from surgical margins and atypical lobular hyperplasia (ALH). The E-cadherin immunostain demonstrated loss of membranous expression in the ALH, while stains for p63 and SMMHC demonstrated retained myoepithelial cells throughout the lesion. Following a discussion regarding treatment options, the patient underwent a right partial mastectomy and axillary sentinel lymph node biopsy, with adjuvant radiation of the right breast. Pathology from this surgery showed no residual tumor, and no tumor was identified in the two sentinel lymph nodes.

**Figure 1:**
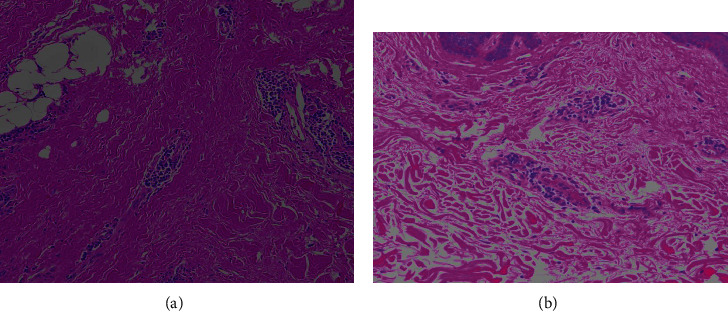
(a) Perivasculitis with edema of the dermis. (b) Perivasculitis with edema of the dermis.

About one month following the completion of adjuvant radiation of the right breast, the patient underwent a thyroidectomy and pathology showed multifocal papillary thyroid cancer. Thyroid ultrasound prior to the thyroidectomy showed enlargement of both lobes, with heterogeneous echogenicity, and was suspicious for small calcification of the lower pole of the right lobe. This was followed by radioactive iodine therapy. About two and a half weeks later, she noticed edema, mild pain, and erythema of the right breast. The patient had previously experienced significant right breast skin changes as a result of her adjuvant breast radiation, including a dermatitis and skin hyperpigmentation; however, the radiation dermatitis had healed prior to the radioactive iodine therapy. We suspected the patient's new symptoms were due to radiation recall dermatitis. However, we decided to evaluate the patient for possible inflammatory breast carcinoma, and incisional biopsies of the right breast were performed. Pathology revealed moderate chronic perivasculitis with edema of the dermis and no evidence of malignancy (Figures 1(a) and 1(b)). The patient was closely monitored, and her symptoms were noted to have improved on subsequent evaluation.

## 3. Discussion

RRD was first described in 1959 in a patient following the administration of dactinomycin [1]. Since then, many other drugs have been associated with this phenomenon, including 5-fluorouracil, hydroxyurea, vinblastine, methotrexate, adriamycin, capecitabine, nitrofurantoin, etoposide, tamoxifen, bleomycin, melphalan, paclitaxel, docetaxel, gemcitabine, pegylated liposomal doxorubicin, interferon *α*-2b, antituberculous drugs, simvastatin, fluoroquinolones, and azithromycin [1, 4, 5].

Most reports describe the lesions as maculopapular eruptions with erythema, edema, vesicle formation, and desquamation, with the reaction ranging in severity from a mild rash to severe necrosis of the skin, and patients commonly report pruritus or pain [3–5]. The exact pathophysiology of RRD is unknown. Depletion and/or changes in functions of stem cells in the irradiated area, overexpression of transforming growth factor *β*1, expression of inflammatory cytokines, keratinocyte necrosis related to cumulative direct DNA damage and oxidative stress, upregulation of thymidine phosphorylase, and depletion of the endogenous scavenger system have all been proposed as possible underlying mechanisms [3, 7].

Diagnosis typically does not require a biopsy; however, it can be performed when in doubt [3, 4]. Given that there were no prior reports of an association between radioactive iodine and RRD, as well as the fact that inflammatory breast cancer can present with similar symptoms as our patient, we decided to perform a biopsy which ruled out a malignancy.

## 4. Conclusion

This case demonstrates a novel association between radioactive iodine and RRD, and clinicians need to keep this phenomenon in mind when dealing with patients who have received radiation therapy. It is currently not possible to predict which patients will be affected or design treatment regimens to eliminate this risk [3].

There are currently no specific therapies available for RRD. RRD may resolve spontaneously, and close observation is an adequate approach, especially when symptoms are not severe; the role of antihistamines, corticosteroids, or nonsteroidal anti-inflammatory drugs in affecting resolution is unclear [2, 3].
